# Heterologous immunity and antibody-dependent enhancement in respiratory virus infections

**DOI:** 10.1007/s11033-025-11189-5

**Published:** 2025-10-29

**Authors:** Nur Hidayah Nor Isamuddin, Sazaly AbuBakar, Kim-Ling Chin, Nurhafiza Zainal

**Affiliations:** 1 Tropical Infectious Diseases Research and Education Centre (TIDREC), University of Malaya, Kuala Lumpur, 50603 Malaysia; 2 Institute for Advanced Studies, Advanced Studies Complex, Universiti Malaya, Kuala Lumpur, 50603 Malaysia; 3 Department of Medical Microbiology, Faculty of Medicine, Universiti Malaya, Kuala Lumpur, 50603 Malaysia

**Keywords:** Heterologous immunity, Cross-reactivity, Antibody-dependent enhancement, Respiratory virus, Universal vaccine

## Abstract

Respiratory viruses such as influenza viruses and coronaviruses pose persistent and evolving threats to global public health, driven by diverse mechanisms of immune evasion, cross-species transmission, and pandemic potential. Understanding the interplay between heterologous immunity and antibody-dependent enhancement (ADE) is crucial in delineating both protective and pathogenic immune responses following infection or vaccination. This review synthesizes current advances in the molecular and cellular mechanisms underlying virus-agnostic innate defenses, adaptive receptor diversification via V(D)J recombination, and the structural and functional bases of T and B cell cross-reactivity. The dualistic nature of antibody responses is examined in the context of Fc receptor- and complement-mediated ADE, emphasizing the implications for immune protection versus immunopathology. The impact of pre-existing cross-reactive immunity, primed by prior exposures to antigenically distinct viruses or vaccines, is discussed with evidence from the severe acute respiratory syndrome coronavirus 2 (SARS-CoV-2) pandemic and other seasonal respiratory outbreaks. Finally, the review evaluates recent progress and ongoing challenges in universal vaccine development, proposing that the rational harnessing of broad-spectrum and cross-reactive immune mechanisms will be essential for enhancing pandemic preparedness and mitigating the risks associated with immune enhancement phenomena.

## Introduction

Respiratory viruses are a significant public health concern worldwide, causing substantial illness, death and economic losses. These infections primarily target the upper and lower respiratory tracts and are caused by various viruses, including rhinoviruses, influenza viruses, coronaviruses, adenoviruses, enteroviruses and respiratory syncytial virus (RSV). The epidemiology reveals that these infections are most prevalent in children and elderly individuals, with severe cases often resulting in complications such as pneumonia or bronchiolitis [1].

Respiratory virus infections can range from mild upper respiratory infections (URIs) affecting the major airways, pharynx, larynx, sinuses, and nose, to more severe lower respiratory tract infections (LRIs) affecting the lungs and the areas below the vocal cords [2]. URIs, commonly known as the cause of the common cold, are self-limiting conditions characterized by irritation and swelling of the upper airways, often accompanied by a cough [3]. On the other hand, chest discomfort, dyspnea, and fever are more common symptoms of LRIs, including pneumonia, bronchitis and bronchiolitis, which can be more severe [4]. LRIs can be caused by influenza virus, parainfluenza virus, respiratory syncytial virus and coronavirus [5, 6]. Millions of cases of acute respiratory infections (ARIs) are reported worldwide each year, with pneumonia being a major cause of death for children under five, particularly in low- and middle-income nations, and it is also associated with a high rate of hospitalization among older adults [7, 8]. The financial ramifications are also significant, placing a burden on healthcare resources due to the high costs associated with hospitalizations.

Respiratory virus infections have also been known to cause multiple outbreaks and even global pandemics. The world was taken aback when a new pandemic caused by SARS-CoV-2 emerged in 2019. The emergence of this pandemic has caused a global health crisis, with the number of confirmed infected cases surpassing 700 million and the death toll reaching over 6 million individuals worldwide [9]. Other outbreaks caused by respiratory viruses include the Middle East respiratory syndrome (MERS) outbreak, caused by the MERS coronavirus (MERS-CoV), which was first identified in Saudi Arabia in 2012 [10]. According to recent reports, there have been over 2,500 confirmed cases with a mortality rate exceeding 30% for MERS-CoV infections as of October 2024 [11]. In addition, the swine flu pandemic emerged in 2009, triggered by a new strain of the H1N1 influenza virus, which spread rapidly worldwide, resulting in an estimated 1.4 billion cases and between 151,700 and 575,500 deaths within the first year alone [12, 13]. These pandemics have highlighted the urgent need for comprehensive research and surveillance of respiratory viral infections, as understanding their transmission dynamics and potential for outbreaks is crucial for preventing future health crises.

Given the high burden and widespread presence of respiratory viruses, interactions between different viral pathogens and the host immune system are inevitable. A key consequence of these interactions is the development of heterologous immune responses, where prior exposure to one pathogen influences the outcome of subsequent infections with unrelated or antigenically distinct viruses. This review addresses several immunological mechanisms underlying such responses. The discussion begins with virus-agnostic innate immunity and the role of trained immunity in modulating susceptibility and disease severity during respiratory viral infections. This is followed by an examination of the adaptive immune system, emphasizing the molecular processes of T and B cell receptor diversification through V(D)J recombination, as well as the contributions of induced fit, degeneracy in TCR recognition, and molecular mimicry in shaping cross-reactive immunity. Overview of the mechanisms related to heterologous immunity are listed in Table 1. The dual-edged phenomenon of ADE is then analyzed, focusing on Fc receptor-mediated mechanisms and complement activation pathways that may exacerbate infection. Subsequently, the impact of pre-existing cross-reactive immunity on the course of the SARS-CoV-2 pandemic is explored. The final section highlights the significance of advancing universal vaccines, emphasizing how insights from these immune mechanisms can inform the development of broadly protective strategies against current and future respiratory viral pathogens.

**Table 1:** Overview of the mechanisms of heterologous immunity

Mechanism	Description	Examples of respiratory virus infection	Key References
Trained Immunity	Long-term functional reprogramming of innate cells via viral/vaccine exposure.	BCG/mucosal vaccine induces protection against influenza, RSV	[14, 15]
V(D)J Recombination	Somatic rearrangement generating diverse TCR/BCR for broad antigen recognition.	Shared memory T-cell/B-cell clones cross-recognize SARS-CoV-2/influenza	[16, 17]
Induced Fit, TCR Degeneracy	TCR structural adaptation to multiple peptides (polyspecificity).	TCRs cross-react with mutated influenza and SARS-CoV-2 epitopes	[18–20]
Molecular Mimicry	Recognition of similar viral or self-peptides by adaptive receptors.	SARS-CoV-2 TCRs cross-reactive with human/self-proteins, MIS pathogenesis	[21–23]

### Virus-agnostic innate responses

Virus-agnostic innate responses constitute the evolutionarily conserved, non-specific branch of host defense that provides an immediate protective barrier against invading viruses, regardless of their antigenic identity. Unlike adaptive immunity, which specifically tailors its response to distinct viral antigens and generates long-lasting memory, innate immunity relies on broad recognition mechanisms involving pattern recognition receptors (PRRs) such as toll-like receptors (TLRs), RIG-I-like receptors (RLRs), and NOD-like receptors (NLRs) [14]. These receptors detect conserved pathogen-associated molecular patterns (PAMPs), enabling the host to recognize diverse viruses through common molecular markers rather than virus-specific features [15]. Upon viral entry, airway epithelial cells and resident immune cells of the respiratory tract rapidly sense PAMPs, initiating intracellular signaling cascades that culminate in the release of interferons (IFNs), as well as pro-inflammatory cytokines and chemokines [16, 17]. Collectively, these mediators establish an antiviral state within the infected tissue, characterized by the upregulation of interferon-stimulated genes (ISGs), inhibition of viral replication, and induction of apoptosis or autophagy, thereby restricting viral spread during the earliest phases of infection.

Type I interferons (IFN-α and IFN-β) are central to this antiviral response. They act on both infected and neighboring uninfected cells to induce ISGs that interfere with various stages of the viral life cycle, conferring immediate antiviral protection while also reducing the risk of subsequent infections with unrelated pathogens [18]. Evidence of this broad protective effect has been demonstrated *in vitro*, where rhinovirus was found to provide protection against the influenza A virus (IAV) during periods of respiratory virus co-circulation due to its ability to induce IFN-dependent ISG expression, as evidenced by a dramatic 50,000-fold decrease in IAV viral RNA observed five days after rhinovirus inoculation [19]. Similarly, respiratory virus infections can trigger a rapid IFN response that provides brief, nonspecific protection against other viruses, as demonstrated by studies showing that prior influenza B infection induces cross-reactive IFN-γ responses that reduce viral shedding upon exposure to different lineages [20].

Despite their benefits, interferons can also exert detrimental effects when dysregulated. Prolonged or excessive IFN production can lead to chronic inflammatory conditions characterized by continuous immune activation, tissue damage, and increased viral loads, as observed in the severity of disease in 1918 influenza-infected macaques where a maladaptive, excessive innate immune response dominated by interferon dysregulation and sustained inflammation failed to control the virus effectively [21]. Additionally, type I IFN induction during influenza virus infection was found to impairs the function of lung γδ T cells (which produce IL-17) and increases susceptibility to secondary *Streptococcus pneumoniae* infection [22]. Conversely, insufficient or blunted IFN signaling, as reported in severe COVID-19 patients with low or absent IFN-α/β responses, similarly predisposes to uncontrolled viral replication and excessive inflammation [23]. These examples underscore the dual nature of IFNs: a timely and well-regulated IFN response confers antiviral protection, whereas either hyperactivation or suppression contributes to immunopathology and poor outcomes.

Beyond molecular mediators, virus-agnostic innate responses depend on the recruitment and activation of cellular effectors, including natural killer (NK) cells, macrophages, dendritic cells, and innate lymphoid cells. These cells exert rapid cytotoxic, phagocytic, and immunomodulatory functions that restrict viral replication and orchestrate downstream adaptive immunity [24]. Importantly, their activation is not virus-specific but rather broadly triggered by PAMPs shared across respiratory viruses. Robust innate immune activation in the upper respiratory tract, characterized by peaking ISG responses as viral load declines, was shown in SARS-CoV-2 infection models to limit early viral replication and spread, with heterologous induction of ISG responses by unrelated respiratory viruses further accelerating viral clearance and suppressing SARS-CoV-2 propagation in airway epithelial organoids and patients [25]. The effectiveness of this immediate, virus-agnostic defense largely determines disease severity, as a prompt and well-regulated innate response can limit infection before tissue damage occurs, whereas a delayed or excessive response may lead to immunopathology, exemplified by the “cytokine storms” observed in severe cases.

An intriguing extension of virus-agnostic innate responses is their contribution to heterologous immunity, a phenomenon whereby exposure to one pathogen or vaccine leads to altered immunity to a different, unrelated pathogen. Recently, heterologous immunity was attributed mainly to cross-reactive T cells and antibodies; recent research demonstrates that the innate immune system can itself “remember” prior encounters through a process termed trained immunity. Trained immunity refers to the long-term functional reprogramming of innate cells, such as monocytes, macrophages, and NK cells, induced by certain viral components or vaccines [26]. These reprogrammed cells display heightened responsiveness on secondary encounters, even with unrelated viruses, resulting in enhanced production of IFNs and inflammatory mediators. For example, “training” of innate immunity with Bacillus Calmette–Guérin (BCG) vaccination, or exposure to certain viral infections, has been shown to confer partial protection against subsequent and antigenically distinct respiratory viruses, including influenza and RSV [27, 28].

In the context of respiratory infections, the cross-protective role of virus-agnostic innate responses is particularly prominent. The respiratory mucosa is persistently exposed to diverse viral threats, and induction of innate antiviral states during one infection can transiently shield others. For example, a study demonstrated *in vitro* and with clinical data that prior rhinovirus infection robustly inhibited influenza A virus replication in the human airway epithelium, primarily through activation of the interferon response [19]. Similarly, vaccines that stimulate strong innate immune activation like live-attenuated influenza vaccines, or even non-specific inducers of innate immunity, have been shown to reduce the incidence or severity of unrelated viral infections in an experimental study [29]. This phenomenon demonstrates both the breadth and functional importance of virus-agnostic innate mechanisms in shaping population-wide susceptibility to respiratory infections.

### Implications of trained immunity in the protection from infection and disease

The enhanced functions of trained immunity include increased cytokine production, heightened phagocytosis, and improved viral clearance, which collectively reduce viral loads, disease severity, and mortality, especially before adaptive immunity is fully activated. For example, alveolar macrophages exposed to SARS-CoV-2 demonstrate enhanced type I interferon responses and heightened antiviral gene expression when challenged with IAV leading to reduced disease severity and mortality [30]. This non-specific heightened immune readiness is dependent on PRR activation and type I interferon signaling, and it is a key determinant of cross-protective innate immune responses in the lung. The broad, antigen-independent characteristics of trained immunity confer heterologous protection against respiratory pathogens, even before or in the absence of pathogen-specific vaccines. Live attenuated vaccines (like BCG and certain mucosal vaccines) and microbial-derived immunomodulators have demonstrated the ability to induce trained immunity, resulting in cross-protection against a wide array of respiratory pathogens beyond their intended targets. For example, intranasal administration of poly-bacterial immunomodulators has conferred protection against both influenza and unrelated viruses such as vaccinia virus, highlighting the utility of trained immunity in broad-spectrum prophylaxis [31]. These effects provide a rationale for developing vaccines and adjuvants that intentionally leverage trained immunity as a strategy for future pandemics and rapidly emerging respiratory viruses. Despite these protective effects, the consequences of trained immunity can be double-edged. In some cases, excessive or maladaptive innate training may lead to heightened inflammatory states, enhanced allergic risk, or immunopathology, especially if pro-inflammatory responses are not well regulated. For instance, experimental studies using RSV models have demonstrated that early-life RSV infection induces TSLP-mediated, long-term reprogramming of dendritic cells toward a hyperinflammatory and allergic phenotype, rather than promoting antiviral immunity [32]. Moreover, heterologous immune responses must be balanced to avoid interference with optimal adaptive responses or the risk of immune-mediated complications. Therefore, understanding the balance between protective and potentially pathologic trained immunity is crucial for the rational design of immunotherapies or vaccines harnessing this phenomenon.

In summary, virus-agnostic innate responses including trained immunity, form the indispensable first line of antiviral defense through rapid PRR-mediated sensing, IFN-driven antiviral states, cytokine release, and effector cell activation. Their tightly regulated activity not only determines the course of primary infection but also underpins transient and sometimes durable cross-protective effects, thereby shaping susceptibility to diverse respiratory viruses at both individual and population scales. These mechanisms highlight a pivotal, often underappreciated, role of innate immunity not merely as a short-lived, non-specific defense but as a dynamic contributor to heterologous protection and respiratory immune homeostasis.

## Molecular mechanisms of T and B cell receptor repertoire based on V(D)J recombination

The molecular mechanisms underlying the diversity of T and B cell receptor repertoires are rooted in V(D)J recombination, a process essential for the adaptive immune system’s capacity to recognize a vast array of antigens, including those from respiratory viruses [33]. This process is pivotal in shaping the foundation for heterologous immunity, where previous exposure to one pathogen influences immune responses to different and unrelated viruses by creating overlapping receptor specificities that enable cross-reactive immunity in respiratory virus infections. V(D)J recombination is a somatic DNA rearrangement mechanism that occurs in developing lymphocytes within the bone marrow (for B cells) and thymus (for T cells) where each receptor (B cell receptor/BCR and T cell receptor/TCR) possesses variable (V), diversity (D; only in heavy chains and some TCR chains), and joining (J) gene segments that are randomly selected and spliced together to form a unique receptor gene [34]. The recombination activating gene products RAG1 and RAG2 orchestrate this rearrangement by recognizing recombination signal sequences (RSS) flanking each V, D, and J gene segment, and introducing DNA double-strand breaks [35]. The repair of these breaks introduces further diversity through nucleotide addition (by terminal deoxynucleotidyl transferase, TdT), deletion, or palindromic addition at junctions, especially in the complementarity-determining region 3 (CDR3), the primary site for antigen recognition [36]. Once V(D)J recombination produces a functional receptor, developing B and T cells undergo positive and negative selection where receptors that bind self-antigens too strongly are either edited (further recombination), deleted, or rendered anergic to prevent autoimmunity [37]. Successfully selected cells then mature and populate the periphery, carrying a receptor uniquely encoded by the V(D)J process.

Heterologous immunity arises when memory T or B cells generated during infection with one virus cross-recognize and respond to antigens from a different, unrelated respiratory virus, due to structural similarity in their epitopes. This cross-reactivity is only possible due to the vast potential of V(D)J recombination, which by generating such broad repertoires, increases the probability that some receptors can recognize conserved motifs or molecular mimics among diverse respiratory viruses. However, the clonal expansion of cross-reactive memory cells during secondary infection can skew the immune response, potentially narrowing the TCR or BCR repertoire (oligoclonality) and altering immunodominance hierarchies [38]. This may enhance protection against some viruses or, conversely, enable escape by viral variants if the response is too focused on cross-reactive but suboptimal clones.

Multiple studies provide compelling evidence supporting the role of V(D)J recombination–driven receptor diversity in heterologous immunity during respiratory virus infections. In murine models, prior heterologous viral infections such as lymphocytic choriomeningitis virus (LCMV) can activate cross-reactive T cells upon subsequent challenge with vaccinia or other respiratory viruses [39], resulting in altered immunodominance where the TCR repertoire properties may be shaped initially by V(D)J recombination. These studies often pinpoint specific TCRs which expand in response to both pathogens, correlating with disease severity and altered immunodominance hierarchies; moreover, expanded populations of memory T cells with cross-reactive TCRs recognizing both SARS-CoV-2 and influenza A virus epitopes have been identified in humans, with many of these T cells sharing similar CDR3β sequences, demonstrating convergent cross-reactive repertoire formation [38]. Besides that, analysis of B cell repertoires in individuals immunized with SARS-CoV-2 vaccines reveals “convergent” V(D)J recombination where distinct individuals generate similar antibody lineages, including IgG and IgA, capable of neutralizing both SARS-CoV-2 and related coronaviruses [40]. Studies have also documented the response of memory B cells containing rare and cross-reactive BCRs, against SARS-CoV-2 and other coronaviruses in both convalescent and vaccinated individuals, underscoring the critical role of BCR repertoire diversity in mediating heterologous immunity to respiratory viruses. These studies further demonstrate that expansion of narrowly focused, cross-reactive lymphocyte clones can provide partial, though often suboptimal immunity to heterologous infection. For example, research has identified a subset of memory B cells that are cross-reactive since they recognize multiple rhinovirus strains, and during acute infection, these cells migrate into nasal tissue and promptly secrete cross-reactive IgG antibodies locally, aiding in viral clearance [41].

## Induced fit, degeneracy of TCR recognition and molecular mimicry

Induced fit, degeneracy of TCR recognition and molecular mimicry are central to understanding heterologous immunity in respiratory virus infection. These mechanisms explain how T cells recognize and respond to diverse and sometimes unrelated viral antigens, forming the basis for heterologous immunity. The “induced fit” model describes how a TCR changes shape upon encountering a peptide-MHC (pMHC) complex, allowing it to mold itself for optimal binding [42]. Structural studies show that the CDR3 of the TCR, which often contains flexible amino acids like glycine, adapts its conformation to match the presented peptide. This flexibility allows a single TCR to recognize multiple, structurally related pMHC complexes, directly enabling cross-reactivity between unrelated viral peptides found in different respiratory viruses. The ability of TCRs to adopt unique conformations upon peptide binding enables a broadened immune response and underpins the rapid recall responses that characterize heterologous immunity. For instance, structural analyses showed these TCRs adopt different conformations upon binding to distinct mutant peptides derived from viral epitopes, allowing them to accommodate amino acid substitutions within the M1 or NP viral epitopes [43]. This conformational flexibility demonstrates induced fit, where TCRs adjust to bind multiple viral sequences, providing cross-reactive potential against drifted or mutated influenza strains that emerge during seasonal epidemics.

On the other hand, TCR degeneracy describes the phenomenon where a single TCR can recognize multiple distinct but structurally related peptides and this ‘polyspecificity’ arises because TCRs typically interact strongly with only a few key peptide amino acids, while tolerating substitutions at others [44]. Such degeneracy ensures that the immune system is equipped to recognize a vast array of potential pathogens and explains why prior infection with one respiratory virus can influence immunity to subsequent unrelated infections. Degeneracy in TCR recognition has been demonstrated by studies in which individual TCR clones isolated from donors infected with or vaccinated against respiratory viruses display the ability to recognize multiple, distinct viral peptides. For example, CD8+ T cell populations specific to dominant influenza A epitopes have been shown to cross-react with peptides from heterologous viruses, consistent with polyspecificity or degeneracy of the TCR repertoire [45]. These findings were supported by an analyses using high-throughput TCR sequencing to identify TCR clonotypes reactive to conserved and variant SARS-CoV-2 epitopes which revealed that dominant TCR sequences could recognize both wild-type and mutant peptide variants, with several clones demonstrating robust cross-reactivity to multiple epitopes in functional assays [46].

Furthermore, molecular mimicry occurs when different pathogens (or host proteins) exhibit similar amino acid sequences or molecular conformations, enabling TCRs initially primed against one antigen to recognize another [47]. In the context of respiratory viruses, molecular mimicry as a driver for cross-reactive T cell responses and potential immunopathology has been robustly documented. This mechanism allows T cells specific to SARS-CoV-2 to cross-react with conserved regions shared by common cold coronaviruses [48], as well as other unrelated viruses. Such cross-reactivity arises from recognition of structurally or biochemically analogous peptides, constituting molecular mimicry at the TCR interface. Besides that, recent studies have identified short sequence or structural homology between viral proteins and self or heterologous viral peptides. For example, an analysis of SARS-CoV-2 demonstrated molecular mimicry with human proteins and showed that cross-reactive TCR clones isolated from patients with multisystem inflammatory syndrome (MIS) could bind both viral and homologous self-derived peptides, thereby implicating molecular mimicry as a contributor to heterologous antiviral immunity as well as post-infectious immunopathology [49]. This process underpins heterologous immunity, facilitating rapid memory T cell responses upon exposure to novel viruses; however, it also carries potential risks by predisposing to immunopathology or autoimmunity if self-peptides are mimicked.

## Antibody-dependent enhancement (ADE)

Antibody-dependent enhancement (ADE) is a multifaceted immunological phenomenon in which an immune response generates antibodies, often pre-existing nonneutralizing or subneutralizing that enhance viral entry, promote replication, and exacerbate infection [50, 51]. This mechanism is particularly evident in patients previously infected with MERS-CoV, who exhibit ADE against SARS-CoV-2, which correlates significantly with low levels of neutralizing antibodies [52]. ADE comprises two distinct mechanisms; extrinsic ADE and intrinsic ADE [53]. Extrinsic ADE involves the uptake of virus-antibody complexes by immune cells (e.g., monocytes and macrophages) via Fc gamma receptors (FcγRs), leading to increased viral entry and a higher number of infected cells [54]. In contrast, intrinsic ADE modulates intracellular immune responses including suppression of IFN and promotes cytokine production, which biases the immune response and amplifies viral replication within infected cells [55, 56]. ADE is also known to be mediated by either Fc receptor or complement activation pathways which will be further elaborated below and summarized in Table 2.

### Fc receptor-mediated enhancement

The mechanism of Fc receptor-mediated uptake in the context of ADE involves interactions between virus-antibody complexes and Fc receptors (FcRs) on immune cells [57]. When antibodies bind to viral particles, it forms immune complexes that can be recognized by FcRs, particularly FcγRs, expressed on the surface of various immune cells such as macrophages and dendritic cells [58]. This binding facilitates the internalization of the virus-antibody complex through a process known as receptor-mediated endocytosis [59]. Once internalized, if the antibodies are nonneutralizing or present at subneutralizing concentrations, the virus can escape degradation and replicate within the immune cell, leading to increased viral loads and enhanced disease severity [60]. Moreover, a study described that changes in antigen presentation by lung epithelial cells could skew antibody responses toward producing nonneutralizing antibodies [61]. These nonneutralizing antibodies can form immune complexes that activate Fc receptors, leading to excessive inflammation and airway obstruction, a phenomenon previously observed in vaccine trials associated with enhanced respiratory disease caused by RSV infection [62]. Conversely, a study demonstrates that neutralizing monoclonal antibodies (MAbs) targeting the receptor-binding domain (RBD) of MERS-CoV and SARS-CoV spike proteins can mediate ADE of viral entry into Fc receptor-expressing human cells, revealing that ADE in coronaviruses can be driven by fully neutralizing antibodies against the same viral strain, unlike flavivirus ADE; which involves subneutralizing or nonneutralizing antibodies and different viral strains, thereby broadening the understanding of ADE mechanisms in respiratory viral infections [63]. In the context of influenza virus infection, antibodies targeting hemagglutinin (HA) have been shown to facilitate ADE by promoting viral entry into immune cells, which results in higher replication rates and severe disease outcomes [64]. Collectively, these findings underscore how Fc receptor-mediated ADE can exacerbate respiratory virus infections by amplifying both viral replication and inflammatory responses.

### Complement system activation enhancement

The complement system plays a significant role in the immune response, assisting in the elimination of pathogens by inducing inflammation and facilitating cell lysis. However, in certain contexts, particularly ADE, this system can inadvertently exacerbate viral replication and disease severity. This duality is particularly evident with the complement component C1q, having opposing effect depending on binding sites on the virus-antibody complexes. For example, C1q treatment differentially modulated IAV replication; it reduced H1N1 replication but significantly increased H3N2 viral transcripts in A549 cells due to the differences in C1q binding sites on HA or NA [65]. Once the virus-antibody complexes are internalized, these complexes can promote viral replication through various mechanisms, including the modulation of intracellular signaling pathways such as suppression of lipopolysaccharide-induced antiviral transcription factor, that favor viral survival and replication [66]. For example, in HIV infections, complement activation has been shown to increase viral entry by enhancing the binding of opsonized virions to cells expressing complement receptors [67]. This phenomenon is not limited to HIV where studies on other viruses, such as SARS-CoV-2, have demonstrated that complement-mediated ADE can lead to excessive immune activation and inflammation, ultimately worsening disease outcomes [68].

Complement-mediated ADE has been observed in respiratory virus infections, particularly with viruses such as RSV and coronaviruses such as SARS-CoV and SARS-CoV-2. For example, studies have shown that nonneutralizing antibodies against RSV can lead to enhanced disease severity through complement activation, where immune complexes formed by antibodies bind to the virus deposit in lung tissues, triggering complement activation and resulting in inflammation and tissue damage [53]. Research has indicated that complement activation can exacerbate COVID-19 severity by promoting the formation of antibody-antigen complexes that activate immune cascades in lung tissue, leading to acute respiratory distress syndrome (ARDS) [58]. Additionally, elevated levels of complement components, such as C3a, have been associated with severe outcomes in COVID-19 patients, highlighting the role of complement-mediated ADE in respiratory infections [69].

**Table 2:** Summary of ADE mechanisms across respiratory viruses

Type	Definition/Key Steps	Virus Examples	Pathways Involved	References
Extrinsic ADE (commonly FcR-mediated)	FcR-mediated uptake of virus-Ab complexes by immune cells.	SARS-CoV-2, MERS-CoV, influenza	FcγR, endocytosis	[54, 63, 64]
Intrinsic ADE	Intracellular immune modulation, cytokine enhancement.	SARS-CoV-2, RSV	IFN suppression, cytokine bias	[53, 55, 56]
Complement-Mediated	Immune complex activation of complement pathways, promoting viral replication and inflammation.	SARS-CoV-2, RSV, influenza	C1q, C3a, complement system	[65, 68, 69]

## Impact of pre-existing cross-reactive immunity on the trajectory of the SARS-COV-2 pandemic

Pre-existing cross-reactive immunity, primarily generated through exposures to common cold coronaviruses, has significantly shaped both individual outcomes and the broader course of the SARS-CoV-2 pandemic. Multiple studies have shown that antibodies and T cells targeting seasonal human coronaviruses such as OC43 and NL63 can recognize SARS-CoV-2 epitopes, frequently resulting in measurable heterologous immunity [70]. For instance, individuals with elevated levels of cross-reactive antibodies prior to SARS-CoV-2 exposure, as reported by Lin *et al.* [71], demonstrated impeded induction of new antibody responses after infection, revealing the functional presence of cross-reactive immunity. Similarly, a multicohort investigation in China found that about 2.73% of individuals unexposed to SARS-CoV-2 were seropositive for anti-SARS-CoV-2 antibodies which likely a result of historic exposure to related coronaviruses [72]. Beyond antibody responses, pre-existing cross-reactive T cells, especially CD4+ memory T cells, are strongly associated with milder clinical outcomes, such as reduced symptom severity and shorter illness duration following SARS-CoV-2 infection, thus impacting hospitalization and mortality rates [73, 74]. Indeed, Grifoni *et al.* [75] observed that about half of pre-pandemic control samples showed cross-reactive activation of CD4+ T cells after stimulation with SARS-CoV-2 antigens, whereas Swadling *et al.* [76] demonstrated expansion of pre-existing SARS-CoV-2-specific T cells in healthcare workers with abortive, seronegative infection. This work was subsequently extended by the same group, showing that airway-resident T cells from unexposed individuals could cross-recognize SARS-CoV-2, underlining the potential protective role of mucosal immunity and its relevance for future mucosal vaccine design [76]. More broadly, additional studies employing diverse immunological tools have reinforced the presence of such pre-existing T cells in the blood [77]. Notably, airway-resident T cells from unexposed individuals also exhibit cross-reactivity against SARS-CoV-2, enabling rapid recruitment of protective immunity upon viral encounter [78]. Collectively, these findings underscore that airway and systemic pre-existing cross-reactive T cells constitute a critical determinant of immune control against SARS-CoV-2.

**Fig. 1:**
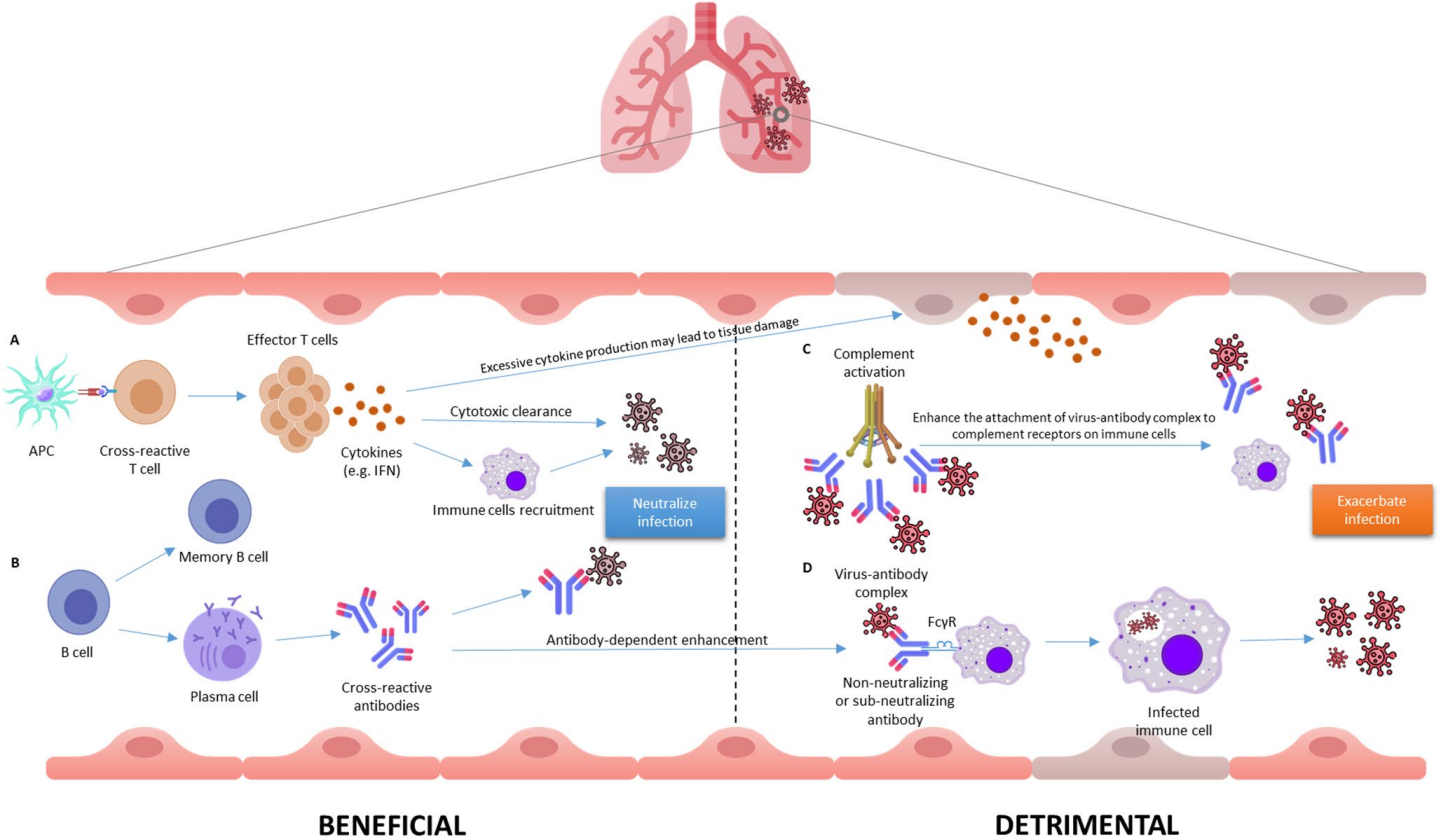
Beneficial and detrimental outcomes of cross-reactive immunity. Beneficial outcomes (A & B) are mediated by effector T cells and cross-reactive antibodies generated from prior antigen exposure, which facilitate cytokine production, cytotoxic clearance, and viral neutralization. Detrimental outcomes (C & D) arise when non-neutralizing or sub-neutralizing antibodies form virus-antibody complexes that can trigger ADE via Fc receptor (FcγR) engagement on immune cells or complement activation, thereby enhancing viral entry, immune cell infection, and immunopathology.

On the other hand, pre-existing cross-reactive immunity can have adverse effects. For example, certain antibody populations may promote ADE, potentially exacerbating disease severity in some settings [58]. Multiple mechanistic studies have shown that infection- or vaccine-induced monoclonal antibodies targeting the SARS-CoV-2 spike protein, particularly those binding the receptor binding domain (RBD) or S1 region, can facilitate viral entry into Fcγ receptor-expressing cells, such as B cells (Raji cells), in an Fc-dependent manner [79]. Clinically, ADE has been detected in serum from a subset of SARS-CoV-2-infected patients (especially those with severe disease) and in individuals vaccinated with adenoviral vector or inactivated SARS-CoV-2 vaccines, but not with mRNA vaccines [80]. In these cases, ADE was associated with reduced neutralizing antibody function and increased complement activation, indicating the potential for non-neutralizing or suboptimal antibody responses to worsen clinical outcomes. Correspondingly, animal and cell line models show that diluted immune sera can augment SARS-CoV-2 replication, whereas high concentrations elicit effective viral neutralization, suggesting a dose-dependent duality to antibody responses [81]. These findings underscore that while cross-reactive immunity can be protective, it may still predispose to ADE or enhanced immunopathology (Fig. 1) during heterologous infection under certain conditions. Thus, highlighting the need for careful immunogen and vaccine design during pandemic response.

## Significance of advancing universal vaccines

Universal vaccines targeting highly mutable respiratory viruses such as influenza and coronaviruses are increasingly vital due to their antigenic variability and pandemic potential. Unlike seasonal vaccines that require continuous reformulation, universal vaccines are designed to elicit durable, broad-spectrum immunity by focusing on conserved viral elements, such as influenza nucleoproteins matrix protein 2 ectodomain (M2e) or the coronavirus S2 domain [82, 83]. These conserved regions can stimulate cross-reactive antibodies and memory T cells, providing heterologous immunity that helps reduce transmission, lessen disease severity, and accelerate protection during outbreaks. Such approaches are particularly valuable for pandemic preparedness, where overlapping circulation of respiratory viruses demands rapid and robust immunity.

Advanced vaccine modalities including mRNA, viral vectors, and protein nanoparticles are being applied to efficiently deliver these conserved antigens, and sequential or combined regimens have demonstrated promise in reinforcing both the breadth and durability of the immune response. Recent research provides multiple examples of how these advanced vaccine platforms have enhanced universal vaccine design. mRNA vaccines encoding conserved internal influenza proteins such as nucleoprotein, matrix protein 1, and polymerase basic protein 1 have consistently induced strong and cross-protective humoral and cellular responses in animal models, conferring protection against a wide array of influenza strains, including those that are antigenically mismatched [84]. Besides that, multivalent mRNA vaccine formulations encoding hemagglutinin antigens from every influenza A and B subtype have elicited long-lasting, cross-reactive antibodies capable of mediating robust protection even in the face of antigenic diversity [85]. Additionally, trials leveraging protein nanoparticles and viral vectors to present multiple conserved antigens, as well as studies combining mRNA or self-amplifying RNA vaccines with inactivated vaccines, have produced broad-spectrum, durable immune responses and enhanced efficacy against heterologous respiratory virus challenge, further supporting the promise of these delivery strategies in the development of universal vaccines [86–88].

Despite these advances, translating broad immunogenicity into reliable protection across populations presents ongoing challenges, including ensuring the immunogenicity of conserved antigens, achieving durable protective responses, scaling production, and meeting regulatory requirements for vaccines with broad claims. The influence of pre-existing cross-reactive immunity further complicates outcomes. For instance, pre-existing CD4+ memory T cells were shown to accelerate protective responses after primary SARS-CoV-2 vaccination, allowing individuals with such immunity to mount more rapid and robust responses than immunologically naïve counterparts [74]. Similarly, vaccination can boost pre-existing cross-reactive antibodies against other human coronaviruses, with elevated responses seen particularly against closely related viruses such as SARS-CoV and MERS-CoV [89]. Moreover, mRNA vaccines such as BNT162b2 have been shown to induce stronger reactivation of cross-reactive humoral and cellular responses compared with adenoviral vector vaccines like ChAdOx1 [90]. Despite these advantages, concerns persist regarding the theoretical risk of ADE, wherein suboptimal antibodies facilitate viral entry through Fc receptor pathways. Although not observed with current COVID-19 vaccines [91], ADE has historical precedent, as demonstrated by the formalin-inactivated RSV vaccine of the 1960s, which predisposed children to more severe pneumonia and higher mortality following RSV infection [92]. These insights underscore that universal vaccine development requires innovative strategies balancing efficacy, safety, and cross-protective potential, while carefully accounting for the complex role of pre-existing cross-reactive immunity in shaping outcomes across diverse populations.

## Conclusion

Collectively, the convergence of insights from innate and adaptive immunology, cross-reactivity, and the risk of ADE underscores the complexity of host–pathogen interactions in respiratory virus infections. While trained innate immunity and broad T and B cell receptor repertoires confer substantial cross-protective benefits, the potential for dysregulated or nonneutralizing antibody responses to amplify disease severity through ADE remains a significant concern in both natural infection and vaccination settings. The multifaceted role of pre-existing immunity shaped by prior infections, vaccination history, and immunogen design, demands ongoing vigilance in pandemic response and vaccine strategy. Advancing universal vaccines that balance efficacy, breadth of protection, and immunological safety, while minimizing the risk of immune-mediated pathogenesis, will be instrumental in combating present and future respiratory viral threats. Sustained interdisciplinary research and continual immunological surveillance are essential to guide the rational design of next-generation immunotherapies and to anticipate the dual-edged consequences of cross-reactivity and enhancing immune responses in diverse populations.

## Data Availability

No datasets were generated or analysed during the current study.

## Abbreviations

ADE: Antibody-dependent enhancement
V(D)J: Variable (V), Diversity (D), and Joining (J) gene recombination
SARS-CoV-2: Severe acute respiratory syndrome coronavirus 2
MERS: Middle East respiratory syndrome
MERS-CoV: Middle East respiratory syndrome coronavirus
RSV: Respiratory syncytial virus
URI: Upper respiratory infection
LRI: Lower respiratory infection
ARI: Acute respiratory infection
PRR: Pattern recognition receptor
TLR: Toll-like receptor
RLR: RIG-I-like receptor
NLR: NOD-like receptor
PAMP: Pathogen-associated molecular pattern
IFN: Interferon
ISG: Interferon-stimulated gene
NK: Natural killer
BCR: B cell receptor
TCR: T cell receptor
RSS: Recombination signal sequence
TdT: Terminal deoxynucleotidyl transferase
LCMV: Lymphocytic choriomeningitis virus
CDR3: Complementarity-determining region 3
FcR: Fc receptor
FcγR: Fc gamma receptor
MAb: Monoclonal antibody
RBD: Receptor-binding domain
HA: Hemagglutinin
NA: Neuraminidase
ARDS: Acute respiratory distress syndrome
M2e: Matrix protein 2 ectodomain
MIS: Multisystem inflammatory syndrome
BCG: Bacillus Calmette–Guérin (vaccine)
